# Dietary Antioxidants in Age-Related Macular Degeneration and Glaucoma

**DOI:** 10.3390/antiox10111743

**Published:** 2021-10-30

**Authors:** Jacek Dziedziak, Kaja Kasarełło, Agnieszka Cudnoch-Jędrzejewska

**Affiliations:** Department of Experimental and Clinical Physiology, Center for Preclinical Research, Medical University of Warsaw, 02-097 Warsaw, Poland; jacek.dziedziak@wum.edu.pl (J.D.); agnieszka.cudnoch-jedrzejewska@wum.edu.pl (A.C.-J.)

**Keywords:** antioxidants, age-related macular degeneration, glaucoma, carotenoids, resveratrol

## Abstract

Age-related macular degeneration (AMD) and glaucoma are ophthalmic neurodegenerative diseases responsible for irreversible vision loss in the world population. Only a few therapies can be used to slow down the progression of these diseases and there are no available treatment strategies for reversing the degeneration of the neural retina. In AMD, the pathological process causes the malfunction and damage of the retinal pigmented epithelium and photoreceptors in the macula. In glaucoma, damage of the retinal ganglion cells and their axons is observed and treatment strategies are limited to intraocular pressure lowering. Therefore, other prophylactic and/or therapeutic methods are needed. Oxidative stress is involved in the neurodegenerative process accompanying both AMD and glaucoma; therefore, the use of antioxidant agents would clearly be beneficial, which is supported by the decreased prevalence and progression of AMD in patients adherent to a diet naturally rich in antioxidants. Dietary antioxidants are easily available and their use is based on the natural route of administration. Many preclinical studies both in vitro and using animal models of retinal degeneration showed the efficacy of dietary antioxidants, which was further proved in clinical trials. Resveratrol is beneficial both in AMD and glaucoma animal models, but confirmed only among AMD patients. For AMD, carotenoids and omega-3 fatty acids were also proved to be sufficient in preventing neurodegeneration. For glaucoma, coenzyme Q10 and alpha-lipoic acid showed efficacy for decreasing retinal ganglion cell loss and inhibiting the accompanying destructive processes. Interestingly, the benefits of vitamins, especially vitamin E was not confirmed, neither in preclinical nor in clinical studies.

## 1. Introduction

The retina of the eye in mammals is a structure consisting of five layers of specialized neurons. Being the neural part of the eye, the retina is responsible for the transduction of external light stimulus to impulses, transmitted along the axons of the retinal ganglion cells to the brain, thus creating visual perception [1,2]. Disturbances in the retinal homeostasis, e.g., intraocular pressure (IOP) elevation, neuroinflammation, increased level of oxidative stress and neural excitotoxicity lead to damage of the retinal cells, neurodegeneration and eventually to blindness [3,4,5].

Retinal neurodegenerative diseases are a rising problem worldwide. The most common are age-related macular degeneration (AMD) and glaucoma [6]. The prevalence of these diseases is increasing and varies worldwide [7,8]. It is estimated that, by 2040, the number of patients with AMD will reach 288 million and with glaucoma over 110 million [9,10]. The neurodegenerative process accompanying both AMD and glaucoma leads to irreversible damage to the neural part of the retina, which is the leading cause of gradual vision loss [11]. As there is no treatment available to reverse degeneration and loss of neurons in the retina, additional and/or supportive therapies are beneficial.

As with other neurons, retinal cells are characterized by high oxygen consumption, which is used for energy production by the oxidation processes within the mitochondria. Reactive oxygen species (ROS), such as superoxide anion (O_2_^•−^), hydrogen peroxide (H_2_O_2_) and the hydroxyl radical (HO^•^) are the standard product of cell metabolism within the mitochondria, but enzymatic reactions and exogenous factors such as smoking, an unbalanced diet (e.g., a western diet), phototoxicity, etc., are sources of ROS [12]. In physiological conditions, ROS are neutralized by a system of antioxidants (e.g., the antioxidative enzymes such as superoxide dismutase or glutathione). In the case of cell metabolism disturbances or antioxidant system insufficiency, an excess of ROS may be produced and unbalanced ROS production generates oxidative stress. During oxidative stress, the oxidation of molecules such as proteins, lipids, carbohydrates and nucleic acid occurs, which may cause their damage and malfunction. Oxidative stress contributes to the damage of cellular structures and cell death. Processes involved are primarily focused on the mitochondria, where excess ROS production leads to (i) a decrease of ATP production in favor of ROS generation, (ii) oxidation of the lipids and proteins, which are the components of the mitochondrial membrane and (iii) DNA oxidation. Next, mitochondrial disruption, energy deficiency, lipid and protein malfunction and cell membrane damage induces cell death [13,14,15,16]. 

The high need for oxygen, the high amounts of polyunsaturated fatty acids and the low antioxidative enzyme levels make the nervous system extremely sensitive to oxidative stress. Additionally, oxidative stress-induced inflammation and blood–brain barrier (BBB) damage contributes to nervous tissue degeneration. The disruption of signal transmission provided by the neurons, i.e., the functional cells of the nervous system, leads to the loss of function. Additionally, the death of glial cells, which are supportive to neurons, also contributes to the neurodegeneration [13,14]. Oxidative stress not only affects the eye and visual processes but also underlies many neurodegenerative diseases such as Alzheimer’s disease (AD), Parkinson’s disease (PD), Huntington’s disease (HD), amyotrophic lateral sclerosis (ALS), Friedreich’s ataxia (FA) and stroke. However, these diseases are beyond the scope of this article [17,18].

In neurodegenerative diseases, dying neurons are replaced by glial tissue, leading to the loss of function of a particular part of the nervous system [19]. Losing neurons of the retina causes vision impairment and blindness; thus, prevention of neuronal death in the retina is crucial. The use of antioxidants would benefit therapy efficiency, regardless of whether the oxidative stress itself is the primary cause of neurodegeneration or the result of ongoing pathological processes underlying the disease.

This review presents the current knowledge of dietary antioxidants which may play a role in diminishing oxidative stress and, therefore diminishing damage to the retinal cells. Use of such dietary agents may be an additional or supportive treatment for neurodegenerative ophthalmic diseases. This review focuses on AMD and glaucoma, which are the two neurodegenerative ophthalmic diseases not arising as complications of systemic disorders. These are the main causes of blindness in developed countries, where oxidative stress is the main factor underlying the pathomechanism [20].

## 2. Age-Related Macular Degeneration

AMD is one of the primary causes of blindness among elderly people in the world. As the population is aging, up to 15–20% may suffer from different stages of AMD. AMD is characterized by the gradual deterioration of functional visual acuity (especially central vision) taking place over many years and eventually leading to irreversible blindness. The first symptoms of AMD are loss of contrast sensitivity, the presence of distorted vision (metamorphopsia) and blurry vision [21]. 

There are several established risk factors for the development of AMD, with age being the major one. Modifiable risk factors are strongly connected to dietary habits—low intake of vitamins A, C and E, zinc, lutein and omega-3 fatty acids [22]. There is also an association between AMD and other systemic disorders. Data from several studies show that there is a link between the occurrence of AMD and hypertension, coronary heart disease, diabetes, dyslipidemia, obesity, high body mass index, chronic kidney disease and AD [23,24,25].

There are two primary forms of AMD: neovascular AMD (nAMD), also referred to as “wet” AMD and “dry” AMD. There is also a subtype of advanced dry AMD known as geographic atrophy (GA). In wet AMD, neovascularization underneath the retinal pigmented epithelium (RPE) occurs, with abnormal blood vessels prone to blood and plasma leakage. Dry AMD is characterized by the presence of drusen, which accumulates between the neural retina and the RPE. In this form, photoreceptors and RPE cell damage is observed [26]. In GA, the degeneration of the retina is profound and no exudates are visible [27]. Although nAMD progresses faster and is more destructive for the retina, there are some therapies available based on the use of anti-VEGF (vascular endothelial growth factor) agents to prevent neovascularization. On the other hand, there is no treatment for dry AMD [28].

RPE is crucial for the survival of retinal cells. RPE cells (i) transport nutrients from blood vessels to photoreceptors, (ii) transport end-products of metabolism back to the blood, (iii) phagocytose the photoreceptor outer segments (POS), which are constantly shed by the rods and cones, (iv) reisomerize all-*trans*-retinal back into 11-*cis*-retinal, (v) produce growth factors and (vi) form the blood-retina barrier. Disturbance of any of those functions may lead to retinal cell death [29].

RPE and photoreceptors are post-mitotic, non-proliferating cells; therefore, the accumulation of damage within the cell and metabolic end-products, etc., increase with age. A high concentration of oxygen due to increased energy demand and irradiation enhances ROS formation and promotes oxidative activity and eventually oxidative stress. RPE and POS are rich in polyunsaturated fatty acids, which are prone to oxidation. This enhances the accumulation of lipofuscin, a product of unsaturated fatty acid oxidation, in RPE cells. Lipofuscin accumulation is mentioned as one of the main risk factors for dry AMD. Those aggregates impede RPE functioning, which further influences the proper functioning of photoreceptors [30,31,32]. The phagocytic activity of RPE, which decreases with age, leads to drusen deposition below the RPE cells, which further induces an inflammatory response [28]. Chronic oxidative stress leading to cell death also causes local inflammation (so-called parainflammation), supporting tissue repair and restoring homeostasis. However, if the oxidative stress is constant, the inflammatory response becomes chronic and causes tissue damage [33,34]. Oxidative stress also enhances angiogenesis, e.g., via ROS derived from NADPH oxidase activity, or via the accumulation of advanced-glycation end products stimulating RPE to VEGF production, promoting neovascularization which is characteristic for wet AMD [35,36].

**Figure 1:**
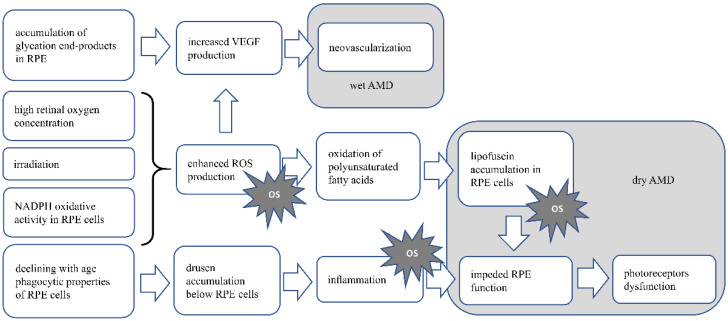
Presents the AMD pathomechanism and oxidative stress involvement.

Figure 1 AMD pathomechanism. In RPE cells and photoreceptors, there is increased oxygen concentration, due to high oxygen demand, supplied by high blood flow in the retina. This along with irradiation leads to enhanced ROS production and possible oxidative stress induction. In dry AMD, the product of oxidation of abundant in retina polyunsaturated fatty acids, the lipofuscin accumulates in RPE cells, leading to their dysfunction, eventually death. As RPE cells are crucial for functioning and survival of photoreceptors, disturbance in their functioning will cause the disturbance in photoreceptor proper functioning and eventually death. Lipofuscin accumulation also causes oxidative stress. Accumulation of drusen below the RPE cells, is the result of declining with age fagocytic abilities of those cells. Drusen accumulation evokes inflammation, which is also the cause of oxidative stress. In wet AMD neoveascularization is induced by VEGF produced by RPE cells. Increased VEGF production may be induced by accumulating end-products of glycation in RPE cells, or by ROS generated in retina. OS—oxidative stress.

### 2.1. Dietary Antioxidants

As there is no treatment available for dry AMD, prevention is the main goal for aging patients. Preclinical and clinical studies support the hypothesis that the use of antioxidants is beneficial in models of oxidative stress-induced ophthalmic diseases. Mediterranean and Oriental diets, naturally rich in antioxidants, were shown to be beneficial for preventing or slowing the progression of AMD in patients [37]. Zinc, resveratrol and carotenoids are substances available in dietary products, which have antioxidative properties and are proven to be beneficial for preventing or slowing the progression of the disease in animal models and in patients with AMD. In vitro studies also showed the protective properties of those substances for RPE and retinal cells. There are mixed outcomes from preclinical and clinical trials about the beneficial role of additional vitamins and omega-3 fatty acid intake.

#### 2.1.1. Zinc

Zinc is a trace element mainly acting as a cofactor for a number of enzymes and is known for its antioxidative properties. Zinc inhibits ROS production by inhibition of NADPH oxidase responsible for O_2_^•−^ generation. It is also the component of superoxide dismutase (SOD), an antioxidative enzyme and inducer of metallothionein, which is the HO^•^ scavenger [38]. Moreover, the nuclear factor erythroid 2-related factor 2 (Nrf2), which increases the expression of antioxidative enzyme (heme oxygenase 1, glutathione, glutathione S-transferase, superoxide dismutase) genes is upregulated by zinc [39,40]. All cell types of the human retina contain ionic Zinc [41].

Preclinical studies in cell cultures revealed that zinc in a dose-dependent manner increased glutathione production in ARPE-19 (the human RPE cell line) cell cultures by activation of the Nrf2-dependent pathway [42]. Zinc also induced the synthesis of antioxidant metallothionein, preventing the lipid membranes from peroxidation. Moreover, it was shown that in Brown Norwegian rats fed with a diet containing insufficient levels of zinc, metallothionein deficiency developed and an increase in lipid peroxidation in retinas was observed [43]. In an animal model of light-induced retinal degeneration developed in Sprague–Dawley (SD) rats, zinc administered intraperitoneally protected the retinal cells from damage [44] and even with increased efficacy when mixed with rosemary extract (*Rosmarinus officinalis*) [45]. 

The role of zinc supplementation in AMD has been verified in several clinical trials. The first clinical trial using orally supplemented zinc was performed by Newsome et al. on a group of 151 patients with AMD or drusen only. There was significantly less visual loss in the treated group than in the placebo group after a follow-up of 12 to 24 months [46]. AREDS (Age-Related Eye Disease Studies) is another large, multicenter, randomized clinical trial, one of the most important in the field of AMD risk factors, with a sample size of 3640 subjects. AREDS proved that supplementation of zinc alone decreases the risk of AMD progression. It also showed that supplementation of zinc and an additional antioxidant supplement significantly reduces the decrease in best corrected visual acuity (BCVA), which is significant in AMD [47]. The outcomes of different studies–the Rotterdam Study, the Blue Mountain Eye Study and the Beaver Dam Eye Study, with cohorts of 4170, 1952 and 1709 people, respectively, confirmed the role of zinc supplementation in suppressing AMD severity progression, especially in the early stages of the disease [48].

#### 2.1.2. Resveratrol

Resveratrol, the phytophenol found primarily in red grapes, red wine and berries, is part of a plant’s anti-fungal defense mechanisms and was proved to have beneficial effects on animal organisms. Resveratrol prevents coronary diseases, has anti-carcinogenic, anti-inflammatory, anti-aging and antioxidative activity [49]. Beneficial effects of the use of resveratrol in AD patients have been widely described [50,51]. For antioxidative effects, resveratrol (i) directly scavenges the free radicals (O_2_^•−^, H_2_O_2_, HO^•^), (ii) activates the modulators of gene transcription, such as SIRT1 (which upregulates the antioxidative enzymes—catalase, superoxide dismutase 2) and Nrf2 and (iii) influences NADPH oxidase activity [52,53].

In in vitro studies, Pintea et al. [54] showed that, in human RPE cell cultures subjected to hydrogen peroxide, resveratrol increased the antioxidative-enzyme levels and decreased ROS production. A similar effect on antioxidative enzyme levels and increased cell viability was observed in RPE cells subjected to stress induced by hydroquinone [55]. Resveratrol also reduced the damage to ARPE-19 cells induced by UVA radiation [56]. N-retinyl-N-retinylidene ethanolamine (A2E) is the component of lipofuscin accumulating in RPE cells. Alaimo et al. [57] showed that resveratrol prevented the damage (mitochondria fragmentation, RPE cell apoptosis) induced by A2E in ARPE-19 cells. Resveratrol also has a potential role in preventing wet AMD. In ARPE-19 cells subjected to oxysterols-induced stress, resveratrol addition inhibited the secretion of VEGF [58]. In another study, ARPE-19 cells were treated with red wine extract containing resveratrol, among other polyphenols, which decreased VEGF-A secretion [59].

There is a limited number of clinical trials with the use of resveratrol. A study performed by Richer et al. on a group of three patients, with the administration of a resveratrol-based drug (Longevinex^®^ which contains 100 mg of resveratrol), showed broad bilateral improvements in the retina and the choroid structure and its function [60]. In another study by Richer, this supplement was also responsible for the anatomic restoration of the retinal structure, the improvement in choroidal blood flow and the general improvement of the RPE function [61]. Ivanova et al. also noted an improvement in both BCVA and retinal thickness in three cases where resveratrol was supplemented [62]. Although follow-up time in these trials and the beneficial effect of the drug were relatively long (at least one year), there is a need for the study to be performed on a greater sample size. 

#### 2.1.3. Carotenoids—Lutein and Zeaxanthin

Lutein and zeaxanthin are the two carotenoids present in the retina, mainly in the fovea, giving the macula its color. They are well-known antioxidants in the eye, due to their scavenging properties for free radicals and, moreover, they can absorb light, which prevents light-induced ROS generation and oxidative stress induction, thus protecting the retinal cell from damage [63,64,65,66]. Lutein and zeaxanthin are abundant in green and orange vegetables, respectively. Both are highly available from egg yolk [67]. The importance of the carotenoids in AD patients has been observed, indicating a lower risk of death in AD patients with higher serum lutein and zeaxanthin levels. Carotenoid supplementation also reduces cognitive dysfunction in AD patients [68,69]. A positive outcome was also observed in ALS patients, with carotenoid supplementation delaying disease onset [70,71].

Many preclinical studies showed the efficacy of carotenoids in the protection of animal retinal cells from oxidative stress. In vitro experiments revealed that ARPE-19 cells subjected to H_2_O_2_-induced oxidative stress showed an increased viability and decreased ROS production when incubated with lutein, but not with zeaxanthin [72]. Recently, the more biologically available form of lutein–the prodrug lutein diglutaric acid was tested. Its greater efficacy than lutein was shown for decreased ROS production, oxidative stress-induced RPE cell apoptosis and increased production of antioxidative enzymes [73]. In experiments conducted using laboratory animals, Thomson et al. [74] proved that both lutein and zeaxanthin given orally to quails prevented apoptosis of the photoreceptors after light-induced retinal damage. Diet supplementation of lutein and zeaxanthin in rhesus monkeys resulted in protecting the retina in the foveal area from blue-light-induced damage compared with animals devoid of dietary carotenoids [75]. In mice with specific deletion of the Sod2 gene in RPE cells, leading to the deficiency of mitochondrial manganese superoxide dismutase and therefore leading to oxidative stress induction, zeaxanthin supplementation showed protective properties. In zeaxanthin-treated mice compared with the control animals, the electrical activity of RPE cells was higher, the expression for antioxidative enzyme genes was increased and the structure of RPE cells was better preserved [76].

The importance of lutein and zeaxanthin was the subject of several clinical trials and observational studies. The first report connecting lutein and zeaxanthin intake with the reduced risk for AMD came from the multicenter Eye Disease Case-Control Study from 1993 [77]. Data from the AREDS study showed that a higher dietary intake of lutein and zeaxanthin was associated with a decreased likelihood of developing large drusen, neovascular AMD or GA [78]. AREDS2, a randomized controlled clinical trial, was introduced to substitute beta carotene from the original AREDS formulation with lutein and zeaxanthin. Beta carotene was removed due to safety issues—its high consumption is a risk factor for lung cancer in smokers. This showed that such intervention could significantly reduce the risk of developing late AMD [79]. However, the primary analysis showed no additional benefits [80]. Recently published post hoc analysis of the AREDS and AREDS2 cohorts emphasized that a high intake of lutein and zeaxanthin significantly lowers the risk of late AMD and nAMD [81]. Another population-based cohort study, the Blue Mountains Eye Study, showed that a higher dietary intake of zeaxanthin and lutein was connected with a reduced risk of developing AMD and nAMD progression [82]. The effect of this diet on advanced AMD risk reduction proved to be long-lasting [83]. The Carotenoids in Age-Related Eye Disease Study (CAREDS) highlighted that a diet with high levels of zeaxanthin and lutein may be beneficial against intermediate AMD [84]. The role of lutein and zeaxanthin supplementation and its potential role in the increase in macular pigment optical density (MPOD), thus giving protection against the onset of AMD, was the aim of the France-based randomized clinical trial. The results of this study showed that 6 months of lutein and zeaxanthin dietary supplementation did not have any impact on MOPD [85]. This is contrary to data from a different clinical trial, the CLEAR study, where lutein supplementation alone for 12 months increased MPOD levels in early-stage AMD patients [86]. This was also true for anthropic AMD patients [87]. The role of supplementation of lutein and zeaxanthin in the enhancement of MOPD among patients with early AMD was also proved in several different clinical trials [88,89,90]. The addition of omega-3 fatty acids to the 12-month-long supplementation of lutein and zeaxanthin in the double-blind, placebo-controlled LUTEGA clinical trial also resulted in an increase in MOPD [91]. The study performed by Fujimura et al. noted a similar impact on MOPD after 6 months of supplementation [92]. A metanalysis by Ma et al. supported the thesis that dietary lutein and zeaxanthin are not associated with a significantly reduced risk of early AMD, but an increased supplementation of these antioxidants may be protective against late AMD [93].

#### 2.1.4. Vitamin E

Vitamin E is abundant in dietary products such as olive oil, canola oil, almonds, hazelnuts, etc. [94,95]. Although vitamin E is known for its antioxidative characteristics, due to its ability to scavenge the peroxyl radicals (ROO^•^), thus inhibiting lipid peroxidation [94,95,96], data indicating vitamin E efficacy in neurodegenerative diseases such as AD, PD, stroke and ALS are inconclusive [97,98]. The results from the preclinical studies of its use for AMD prevention are also indecisive. It was shown that, in the absence of vitamin E in genetically engineered mice, increased lipid peroxidation and degeneration of the retina were observed [99]. However, in Fisher rats subjected to light-induced stress, both in normally-fed animals and animals with vitamin E deficiency, the loss of photoreceptor cells in the retina was noted [100].

There are numerous clinical studies about the role of vitamins in AMD, especially for vitamins A, C and E, but their results are inconsistent. Data from the National Health and Nutrition Examination Survey showed that a high consumption of vegetables and fruits rich in vitamin A decreased the risk of AMD [101]. The high consumption of vitamin C in a diet was not associated with risk reduction. In the USA-based case-control study from 1994, there was no statistically significant risk reduction for AMD among those with a high intake of preformed vitamin A (retinol) nor vitamin E nor vitamin C [77]. These findings were later confirmed in the AREDS study [102]. Data from the AREDS study also showed that a supplement containing not only vitamin C and vitamin E but also beta carotene and zinc reduced the five-year risk of developing AMD by 25 percent in patients at risk [103]. This effect continued for five years after the clinical trial ended [47]. In the post hoc analysis of the AREDS and AREDS2 cohorts, there was a decrease in the risk of late AMD and GA for vitamin A and vitamin C. Vitamin A was also associated with a decreased risk of nAMD [81]. A population-based study on AMD risk factors showed that vitamin E but not vitamin A nor C might protect against AMD [104]. This is in contrast with the study on 1193 healthy women where daily administration of vitamin E at a dose of 500 IU did not prevent the development or progression of AMD [105]. Another research study identified that the dietary intake of vitamin E and vitamin C was associated with a reduced risk of nAMD in elderly patients in Japan [106]. Dietary vitamin A showed no correlation with the risk of nAMD. In the Cochrane Database of Systematic Reviews, the intervention of taking supplements of vitamin C or vitamin E made little or no difference in the chances of developing any AMD with a high certainty in evidence [107] and this was consistent with another metanalysis [108]. The role of vitamins in the prevention or progression of AMD needs to be further explored.

#### 2.1.5. Omega-3 Fatty Acids

Long-chain n-3 (omega-3) fatty acids, abundant in fish and fish oil, exert a protective function for retinal cells via antioxidant activity, probably due to changing the composition (replacement of arachidonic acid) of the cell membrane and thus reducing the possibility of lipid peroxidation [109,110,111]. Supplementation of omega-3 fatty acids has been shown to be beneficial in early stages of both AD and PD [112].

Docosahexaenoic acid (DHA) was tested for its protective role in cultured Wistar rat retinas. Retinas were subjected to paraquat, which has oxidative activity. The addition of DHA to the cell culture decreased photoreceptor apoptosis and the mitochondrial membrane integrity was preserved. Expression of anti-apoptotic Bcl-2 protein was also increased in DHA-treated retinal cultures [113]. However, in SD rats fed with a DHA precursor, linolenic acid, the DHA levels in the photoreceptors was increased and after exposure to light, more significant damage of the retinal and RPE cells was observed in comparison with the control animals, as DHA is prone to oxidation [114].

The role of omega-3 fatty acid supplementation in preventing the onset or inhibiting the progression of AMD was the subject of several clinical trials. According to the results of a multicenter case-control study on a group of 349 people, a higher intake of omega-3 fatty acids was associated with a lower risk for AMD, but only if the diet was low in linoleic acid, an omega-6 fatty acid [115]. The protective effect of dietary intake of omega-3 fatty acids against nAMD was confirmed in the AREDS study [102], as well as their impact on reducing the progression of early and advanced AMD [116]. In the AREDS2 study, the addition of DHA and eicosapentaenoic acid (EPA) to the AREDS formulation (vitamin C, vitamin E, β-carotene, zinc and copper) failed to further reduce the risk of progression to advanced AMD [80]. In the randomized, double-blind, placebo-controlled LUTEGA study, an orally administered supplement containing a combination of lutein, zeaxanthin, DHA and EPA for 12 months improved the plasma antioxidant capacity and MOPD [117]. MOPD was also positively correlated with the plasma levels of DHA and EPA in another observational study [118]. Another cohort study proved a significant association between DHA, EPA intake and a decreased risk of the incidence of AMD in women [119]. The previously mentioned AREDS and AREDS2 cohort analyses highlighted the strong link between DHA and omega-3 fatty acid intake and the decreased risk of nAMD [81]. On the contrary, in a randomized controlled trial by Souied et al., three years of oral DHA-enriched supplementation showed no additional effect on the incidence of choroidal neovascularization (CNV) in the other eye in patients with unilateral nAMD [120]. No significant overall impact on the incidence of AMD or its progression was also observed in the VITAL study, covering the role of supplementation of the marine omega-3 fatty acids in AMD [121]. Further research may result in vital new insights.

The outcomes of clinical trials and case series in which the role of supplementation of various antioxidants on AMD was studied are presented in Table 1.

## 3. Glaucoma

Glaucoma is a group of progressive neuropathies of the optic nerve. The underlying causes of glaucomatous neurodegeneration are a significant loss of retinal ganglion cells (RGCs), whose axons form the optic nerve and a thinning of the retinal nerve fiber layer (RNFL), leading to irreversible visual impairment [44,122]. The number of patients with glaucoma is estimated to be greater than 70 million worldwide, with the primary open angle glaucoma being its most common form [123]. There are several risk factors for glaucoma development with excessive caffeine intake, cigarette smoking and poor dietary antioxidants intake being the most important modifiable risk factors [124].

The pathomechanism of open angle glaucoma is mainly based on elevated IOP, which presses against the axons of the RGC, impeding axonal transport, thus leading to the death of the neurons and optic nerve damage causing vision loss [125]. Mechanical stress, aging and accumulating damage in the cells are the basis for mitochondria dysfunction, which results in increased ROS production and oxidative stress generation [126]. Elevated IOP also influences blood circulation in the eye [125]. The altered blood flow causes transient ischemia, followed by reperfusion and during reperfusion of the ischemic tissue, oxidative stress is generated. This occurs mainly in the mitochondria, which are abundant in the retinal neurons due to high energy demand. This may lead to great oxidative stress generation [127]. Oxidative stress is the main factor leading to the death of RGCs. Generated reactive oxygen and nitric species, activation of caspase-3 and peroxidation of polyunsaturated fatty acids are the apoptotic signals for RGCs [128]. 

There are two types of primary glaucoma. The first type is the closed angle glaucoma, which is less frequent and is caused by a rather sudden total blockage of the aqueous humor flow. In this case, laser or surgical intervention is needed. The second type is primary open angle glaucoma (POAG). In this case, the pathological changes progress slowly over the years. Before surgical treatment is considered in this case, therapy is based on lowering IOP, which is achieved by decreasing aqueous humor production or increasing aqueous humor drainage; therefore, efficient non-invasive therapies, such as supplementation with dietary antioxidants would be beneficial [125,129]. 

**Figure 2:**
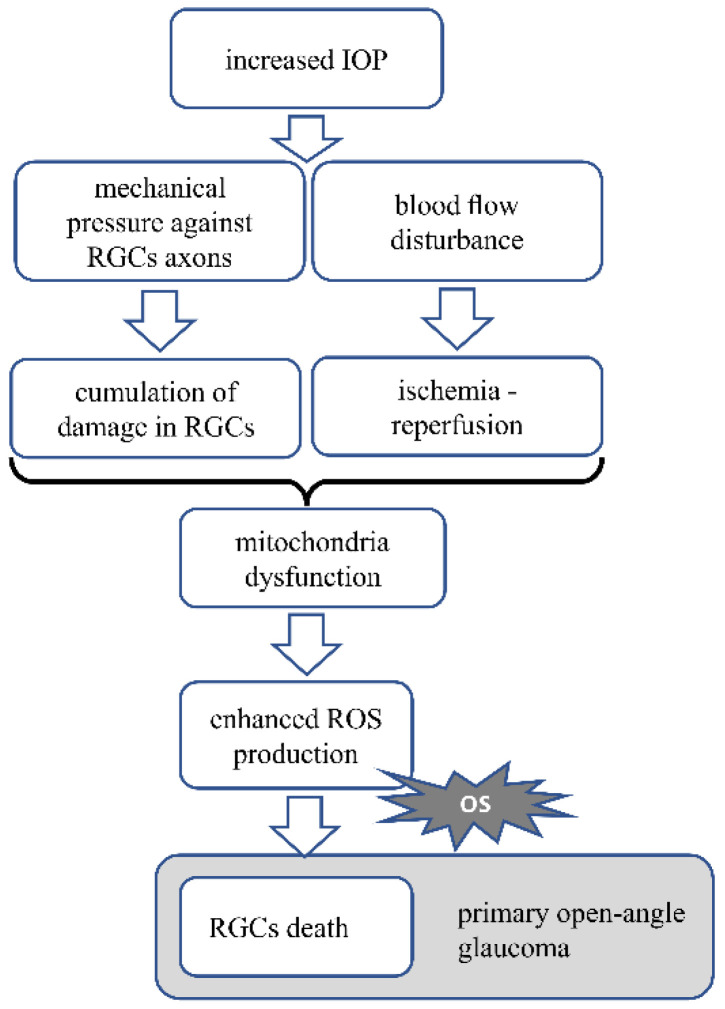
Presents the AMD pathomechanism and oxidative stress involvement.

Figure 2 Pathomechanism of glaucoma. In glaucoma, increased IOP press against the axons of RGC. Mechanical stress and accumulating damage in the cells cause mitochondrial dysfunction, resulting in increased ROS production and oxidative stress generation. Increased IOP also disturbs the blood circulation, causing transient ischemia followed by reperfusion, which generates oxidative stress. Oxidative stress is the main factor leading to RGCs death. OS—oxidative stress.

### 3.1. Dietary Antioxidants

Less data are available about the role of dietary antioxidants in glaucoma than in AMD, especially in clinical studies. Unlike AMD, where oxidative stress is the key factor inducing the disease, glaucoma is a disease of elevated IOP, where sequential changes lead to oxidative stress generation. Nevertheless, whenever oxidative stress is generated, the supplementary use of antioxidants would be beneficial [30,32,126].

#### 3.1.1. Resveratrol

In in vivo studies, results are consistent with the hypothesis that resveratrol is beneficial in glaucomatous conditions in the eye. Resveratrol given orally prevented RGCs from losing dendrites in YFP (yellow fluorescent protein, background C57BL/6) mice subjected to optic nerve injury [130]. Intragastrically administered resveratrol in SD rats subjected to chronic ocular hypertension induced by the injection of superparamagnetic iron oxide into the anterior chamber of the eye resulted in increased cell density in the ganglion cell layer of the retina and decreased apoptosis, compared with the control. Moreover, in the RGC-5 cell line, cultured under elevated pressure conditions, resveratrol reduced the apoptosis of RGC-5 cells and ROS production. In addition, the positive effect on the mitochondria was shown, with decreased markers of mitochondrial dysfunction such as mitochondria membrane depolarization, mitochondria swelling and quantity [131]. The effect of resveratrol administered intraperitoneally was tested in Wistar rats with experimental glaucoma induced by injection of hyaluronic acid into the anterior chamber of the eye. Results showed that resveratrol delayed the loss of RGCs [132]. Intraperitoneal administration of resveratrol was also beneficial in C57BL/6J mice with retinal ischemia/reperfusion injury induced by elevated IOP by saline injection into the anterior chamber. In this study, treatment with resveratrol resulted in a decreased loss of retinal cells and downregulation of the expression of caspase-3 and caspase-8, thus inhibiting apoptosis [133]. Similar results were obtained by Luo et al. [134], where intraperitoneally delivered resveratrol prevented RGC loss and decreased Bax and caspase-3 levels in the retina of SD rats. 

No human glaucoma studies are investigating the role of resveratrol administration.

#### 3.1.2. Coenzyme Q10

Coenzyme Q10 (CoQ10) is an element of the electron transport chain in the mitochondria and is directly responsible for the redox state in the mitochondria. CoQ10 acts as a free radical scavenger [135,136]. CoQ10 is present in plant and animal cells and is available in both animal and plant-derived food. The positive effects of CoQ10 supplementation on some aspects of a number of neurodegenerative diseases, mainly PD, was indicated; however, some results are inconclusive [137,138,139].

Focusing on the retina, in vitro studies showed that CoQ10 decreased the death of cultured RGCs subjected to oxidative stress induced with H_2_O_2_ [140]. Although many in vivo studies focused on the topical administration of CoQ10 [141,142], there are also studies showing its antioxidant activity as a dietary supplement. Lee et al. [143] tested the effect of a diet supplemented with CoQ10 in the spontaneous model of glaucoma in DBA/2J mice. The results showed that in glaucomatous mice CoQ10 supplementation promoted the survival of RGCs and their axons myelination. Additionally, the decrease in expression of GFAP, a marker of astroglial activation was observed. CoQ10 supplementation in DBA/2J mice also reduced the expression of pro-apoptotic Bax protein compared with the control animals. Similar results were obtained for the reduced form of CoQ10–ubiquinol. Ju et al. [144] observed a more remarkable survival of RGCs, decreased astroglial activation and decreased Bax protein expression in C57BL/6J mice subjected to transient retinal ischemia, fed with a diet supplemented with ubiquinol. In another study, paraquat was administered additionally into the vitreous humor of DBA/2J mice to induce oxidative stress. There was also promotion of the survival of RGCs and a decrease in BAX activation observed [145].

Several studies examined the topical administration of CoQ10 and vitamin E eye drops in glaucoma. Such intervention proved to be beneficial for patients with OAG, as it improved the electrophysiological parameters of the inner retina and the optic nerve [146]. A randomized prospective clinical trial from 2019, measuring the impact of the use of topical CoQ10 and vitamin E on the levels of oxidative stress markers in eyes with pseudo-exfoliative glaucoma, showed a significantly lower level of superoxide dismutase in the aqueous humor of treated patients [147]. The study protocol in which this particular formulation was to be assessed in a group of patients with POAG was published in the same year. A total of 612 patients was to be enrolled [148].

#### 3.1.3. Vitamin E

Results from preclinical studies including vitamin E as an antioxidative agent in glaucoma models are not conclusive, pointing to the engagement of vitamin E deficiency in the pathology of glaucoma rather than to the positive benefits from the additional supplementation. 

Wistar rats with elevated IOP evoked by cauterization of three episcleral veins were fed with a control diet or a vitamin E deficient diet or a diet supplemented with vitamin E. The results showed that although vitamin E deficiency caused an increased loss of RGCs in rats with elevated IOP, vitamin E supplementation has no beneficial effect on the survival of RGCs. In vitamin E deficient rats, lipid peroxidation was increased, the activity of superoxide dismutase and catalase were unchanged and the glutathione level was increased [149]. The subcutaneous administration of vitamin E to guinea pigs subjected to a retinal ischemia-reperfusion event resulted in protection against inner plexiform layer edema [150].

There is no information about any clinical trial mainly focusing on the antioxidant properties of vitamin E and their role in glaucoma.

#### 3.1.4. Alpha-Lipoic Acid

Alpha-lipoic acid (ALA) is a natural antioxidant synthesized in many tissues and available in the diet from vegetables and animal tissues, especially the liver and heart. Alpha-lipoic acid antioxidative activity is due to its ability to scavenge free radicals and increase the glutathione level [151,152,153]. Alpha-lipoic acid has been shown to exert a protective role for memory and cognitive function preservation in AD patients [154].

In vitro studies revealed that R-alpha-lipoic acid (R-LA) exerts an antioxidative effect in the RGC-5 cell line due to increased heme oxygenase-1 (HO-1) expression via the Keap1/Nrf2 signaling pathway. In RGC-5 cells subjected to oxidative stress induced by the addition of H_2_O_2_, R-LA decreased RGC-5 cell death. Moreover, intravitreal administration of R-LA to C57BL/6 mice subjected to optic nerve crush resulted in RGC protection and HO-1 upregulation [155]. ALA beneficial activity for glaucoma therapy was tested in DBA/2J mice developing spontaneous glaucoma, fed with a diet supplemented with ALA. ALA treatment was protective for the RGCs and their axons in DBA/2J mice. ALA also presented antioxidative activity while lipid peroxidation was decreased and Nrf2 and antioxidative enzyme (glutathione-S-transferase, glutathione peroxidase 4, peroxiredoxin 2) expression was increased following ALA treatment [156]. Intraperitoneal administration of ALA also decreased RGC death in Wistar rats subjected to ischemia-reperfusion retina injury [157].

The only clinical trial covering the supplementation of vitamin formula enriched with DHA and ALA over a six-month period showed a significant increase in the plasma total antioxidant status in the POAG group [158]. The next step should be an assessment of the ALA intake and its correlation with the parameters describing the advancement of glaucoma.

#### 3.1.5. Omega-3 Fatty Acids

There are few clinical trials about the role of omega-3 fatty acid intake in glaucoma. In an open-label randomized controlled clinical trial, there was no beneficial effect of omega-3 fatty acids on the treatment of mild/moderate POAG. Moreover, in this trial, patients received oral antioxidant supplementation consisting of vitamins A, C and E, lutein, zeaxanthin, zinc, copper, selenium and manganese and this also did not show any significant improvement [159]. In another prospective, randomized, open-label study, the six-month-long administration of a highly rich DHA supplement showed IOP reduction and improvement of oxidative stress parameters among a group of patients with glaucoma secondary to pseudoexfoliation syndrome [160]. There is not much data in this field, so further research is needed.

#### 3.1.6. Hesperidin

Hesperidin is a plant derived flavonoid found in high concentration in citrus fruits. Hesperidin antioxidative activity is due to the activation of the Nrf2-dependent pathway, leading to the upregulation of gene expression for antioxidative enzymes [161,162]. Hesperidin shows a beneficial outcome from its use in such neurogenerative diseases as AD, PD and HD [163]. 

Preclinical studies showed that hesperidin reduced the mouse retinal cell death in vitro. In mice, intravitreal administration of hesperidin reduced the RGC death following NMDA-induced excitotoxic retinal damage [164]. 

There is only one clinical study covering the role of hesperidin on oxidative stress in normal tension glaucoma. Daily oral supplementation of hesperidin enriched with crocetin and *Tamarindus indica* was effective in lowering the oxidative stress level among patients with a high oxidative stress level [165].

Table 2 presents the effects of clinical trials focused on the use of the abovementioned dietary antioxidants on glaucoma.

## 4. Discussion 

AMD and glaucoma are the primary neurodegenerative ophthalmic diseases, where damage of the retinal cells is observed. In AMD, the involvement of the RPE cells in disease pathogenesis is crucial. RPE cells are responsible for the photoreceptor function and survival and as they are prone to homeostasis disturbances, they may easily affect photoreceptor cells. Zinc, resveratrol, omega-3 fatty acids, lutein and zeaxanthin exert a protective effect on RPE cells in in vitro studies by increasing the levels of antioxidative enzymes and increasing the survival rate of the cells. The increased survival rate of retinal cells was confirmed in animal studies.

In glaucoma, elevated IOP directly damages the RGCs by pressing against their axons. It also indirectly promotes ischemia-reperfusion events by affecting the blood flow, which results in the oxidative stress-induced death of RGCs. Resveratrol, coenzyme Q10 and alpha-lipoic acid in in vitro and in vivo studies promotes the survival of RGCs. Alpha-lipoic acid and hesperidin activates also the Nrf2-dependent pathway leading to antioxidative enzyme production.

Interestingly, both in AMD and glaucoma in vitro and in vivo studies, the results obtained for vitamin E administration were questionable.

Clinical trials conducted in both AMD and glaucoma patients showed promising results indicating the effectiveness of the use of most of the presented dietary antioxidants to slow down or prevent disease progression.

Nevertheless, the outcomes of several clinical trials are inconsistent, especially those mainly focusing on the role of vitamins and omega-3 fatty acids in glaucoma and AMD, so there is still a need for large, properly planned studies in this area. Studies covering the role of alpha-lipoic acid, resveratrol and hesperidin in glaucoma and resveratrol in AMD will be of great importance, as studies on animal models or case series showed promising effects of such interventions.

## 5. Conclusions

Oxidative stress is involved in the neurodegenerative process underlying both AMD and glaucoma and therefore the use of antioxidants appears to be beneficial. Dietary antioxidants are naturally available and their route of administration is convenient. For AMD, there is treatment available only for its more advanced, neovascular form. There are no known therapies for dry AMD and GA. In glaucoma, treatment strategies are based on reducing the IOP. There is an emerging need for other prophylactic and therapeutic options, as the number of patients with these diseases is constantly growing. In this paper, we summarized the data from preclinical and clinical studies about the use of dietary antioxidants in animal models and patient groups suffering from AMD or glaucoma. Most of the analyzed antioxidants, such as resveratrol, carotenoids or coenzyme Q10, proved their beneficial effects in preventing retinal cell neurodegeneration. Their use in clinical trials was characterized by a very good safety profile, with hardly any adverse effects of the therapies. In addition, the fact that populations adherent to a diet naturally rich in antioxidants, such as the Mediterranean or Oriental diets, present decreased prevalence and progression of AMD compared with those populations on a Western diet, strongly supports the hypothesis of the beneficial use of dietary antioxidants in the prevention and treatment of neurodegenerative ophthalmic diseases.

## Figures and Tables

**Table 1:** The effects of antioxidant supplementation on the incidence and progression of AMD in clinical trials and case studies.

Antioxidant	Authors	Year of Publication	Population	Study Type	Intervention	Main Outcome(s)	Citation
Zinc	Newsome et al.	1988	151 patients with AMD or drusen only	Prospective, randomized, double-blinded, placebo-controlled trial	Oral supplementation with Zinc sulfate (100 mg) twice a day	Less visual loss then the placebo group after follow up of 12 to 24 months	[46]
Zinc	Chew et al.	2013	3549 patients with varying severity of AMD	Multicenter, randomized controlled clinical trial, followed up by an epidemiological follow up study	Supplementation with antioxidants C, E and β-carotene and/or zinc	Supplementation of zinc only decreased the risk of AMD progression. Supplementation of zinc and other antioxidants significantly reduced the decrease in BCVA	[47]
Resveratrol	Richer et al.	2014	3 patients with AMD	Case reports	1 capsule of Longevinex^®^ (contains 100 mg of RSV) per day	Bilateral improvements in the retina and choroid structure and function	[60]
Resveratrol	Richer et al.	2013	3 patients with AMD	Case reports	1 capsule of Longevinex^®^ (contains 100 mg of RSV) per day	Restoration of retinal structure, improvement in choroidal blood flow and improvement of RPE function	[61]
Resveratrol	Diyana et al.	2017	3 patients with AMD	Case reports	1 capsule Longevinex^®^ (contains 100 mg of RSV) per day	Improvement in both BCVA and retinal thickness	[62]
Lutein and zeaxanthin	SanGiovanni et al.	2007	4203 participants at risk for developing late AMD	Randomized, controlled clinical trial	Supplementation of vitamin C, vitamin E, zinc and lutein/zeaxanthin	Risk reduction for developing late AMD	[78]
Lutein and zeaxanthin	Korobelnik et al.	2017	120 patients without any form of AMD	Randomized clinical trial	Supplementation containing lutein, zeaxanthin, omega-3 fatty acids	No beneficial effect on MOPD	[85]
Lutein and zeaxanthin	Murray et al.	2013	72 patients with early AMD	Randomized, double-blinded, placebo-controlled clinical trial	Daily supplementation with lutein capsules	Lutein supplementation increased MPOD levels in early stage AMD patients	[86]
Lutein and zeaxanthin	Richer et al.	2004	90 patients with atrophic AMD	Prospective, randomized, double-blinded, placebo-controlled clinical trial	Daily supplementation with lutein alone or lutein and other antioxidants	Visual function was improved with lutein alone or lutein together with other nutrientsantioxidants	[87]
Lutein and zeaxanthin	Ma et al.	2012	108 patients with early AMD	Randomized, double-blinded, placebo-controlled clinical trial	Supplementation with lutein and/or zeaxantin	In patients with early AMD, supplementation with lutein and zeaxanthin improved macular pigment density	[88]
Lutein and zeaxanthin	Huang et al.	2015	112 patients with early AMD	Randomised, double-blinded, placebo-controlled clinical trial	Supplementation with lutein and/or zeaxantin	Supplementation with lutein and/or zeaxanthin increased MPOD	[89]
Lutein and zeaxanthin	Akuffo et al.	2015	67 patients with early AMD	Randomised, double-blinded, placebo-controlled clinical trial	Supplementation with lutein and zeaxantin	Supplementation with lutein and/or zeaxanthin increased MPOD	[90]
Lutein and zeaxanthin	Dawczynski et al.	2013	172 patients with non-exudative AMD	Double-blinded, placebo-controlled clinical trial	Supplementation of lutein and zeaxanthin and omega-3-fatty acids	Supplementation caused an increase of MPOD, an improvement and stabilization in BCVA in AMD patients	[91]
Lutein and zeaxanthin	Fujimura et al.	2016	20 patients with nAMD or chronic central serous chorioretinopathy	Clinical trial	Supplement with Lutein, zeaxantin and DHA	Increase in foveal MPOD	[92]
Vitamins	Age-Related Eye Disease Study Research Group	2001	3640 patients with different stages of AMD	Randomized, placebo-controlled, clinical trial	Supplementation with high-dose vitamins C and E, beta carotene and zinc	It reduced the risk of developing AMD and advanced AMD	[103]
Vitamin E	Taylor et al.	2002	1193 healthy participants	Prospective, randomized placebo-controlled clinical trial	Vitamin E 500 IU daily	Daily supplement with vitamin E did not prevent the development or progression of early or later stages of AMD	[105]
omega-3 fatty acids	Chew et al.	2014	4203 patients at risk for progression to advanced AMD	Multicenter, randomized, double-blinded, placebo-controlled clinical trial	Daily lutein, zeaxanthin, DHA and EPA supplementation	Addition of lutein and zeaxanthin, DHA and EPA, or both to the AREDS formulation did not further reduce risk of progression to advanced AMD	[79]
omega-3 fatty acids	Arnold et al.	2013	172 patients with dry AMD	Randomized, double-blinded, placebo-controlled clinical trial	Daily lutein, zeaxanthin, DHA and EPA supplementation	Improvement of plasma antioxidant capacity, circulating macular xanthophyll levels and the MPOD	[117]
omega-3 fatty acids	Souied et al.	2013	263 patients with early lesions of AMD	Randomized, placebo-controlled, double-blinded, comparative study	Daily supplementation with DHA and EPA	No beneficial effect on nAMD incidence	[120]
omega-3 fatty acids and vitamin D	Christen et al.	2020	25871 participants	Randomized clinical trial	Daily supplementation of vitamin D3 and omega-3 fatty acids	Neither vitamin D3 nor omega-3 fatty acids supplementation had a significant effect on AMD incidence or progression	[121]

**Table 2:** The effects of antioxidant supplementation and therapies on glaucoma in clinical trials/case studies.

Antioxidant	Authors	Year of Publication	Population	Study Type	Intervention	Main Outcome(s)	Citation
Coenzyme Q10	Parisi et al.	2014	43 patients with open angle glaucoma	Clinical trial	Topical administration of CoQ10 and vitamin E	Beneficial effect on the inner retinal function and optic nerve electrophysiological parameters	[146]
Coenzyme Q10	Ozates et al.	2019	64 pseudophacic patients (also with pseudo-exfoliative glaucoma)	Prospective, randomized clinical trial	Topical administration of CoQ10 and vitamin E	Lower level of superoxide dismutase in the aqueous humor of treated patients	[147]
Alpha-lipoic acid	Sanz-González et al.	2020	30 participants with and without open angle glaucoma	Prospective clinical trial	Supplementation with formulations containing antioxidant vitamins, alpha-lipoic acid and docosahexaenoic acid	Significant increase in the plasma antioxidant status	[158]
Omega-3 fatty acids	Garcia-Medina et al.	2015	117 patients with mild or moderate primary open angle glaucoma	Open-label, randomized controlled clinical trial	Oral antioxidant supplementation with and without omega-3-fatty acids	No beneficial effect of omega-3 fatty acids on the treatment of mild/moderate primary open angle glaucoma	[159]
Docosahexaenoic acid	Romeo-Villadóniga et al.	2018	47 patients with pseudoexfoliative glaucoma	Prospective, randomized, open-label study	Supplement rich in docosahexaenoic acid	IOP reduction and improvement of oxidative stress parameters	[160]
Hesperidin	Himori et al.	2021	30 patients with normal tension glaucoma	Prospective study	Daily oral supplementation of hesperidin, croce-tin, and *Tamarindus indica*	antioxidant supplementation was effective in lowering oxidative stress level in patients with high oxidative stress level	[165]
